# Hereditary Angioedema Due to C1 Inhibitor Deficiency in Serbia: Two Novel Mutations and Evidence of Genotype-Phenotype Association

**DOI:** 10.1371/journal.pone.0142174

**Published:** 2015-11-04

**Authors:** Slađana Andrejević, Peter Korošec, Mira Šilar, Mitja Košnik, Radovan Mijanović, Branka Bonači-Nikolić, Matija Rijavec

**Affiliations:** 1 Clinic of Allergology and Immunology, Clinical Center of Serbia, Belgrade, Serbia; 2 University Clinic of Respiratory and Allergic Diseases Golnik, Golnik, Slovenia; Cancer Research Centre of Lyon, FRANCE

## Abstract

Hereditary angioedema due to C1 inhibitor deficiency (C1-INH-HAE) is a rare autosomal dominant disease characterized by recurrent life-threatening oedemas and/or abdominal pain and caused by mutations affecting the C1 inhibitor gene, *SERPING1*. We sought to investigate the spectrum of *SERPING1* mutations in Serbia and the possible genotype-phenotype association. C1-INH-HAE was diagnosed on the basis of clinical and laboratory criteria in 40 patients from 27 families; four were asymptomatic. Mutational analysis of the *SERPING1* gene was performed by sequencing and multiplex ligation-dependent probe amplification. Disease-causing mutations in *SERPING1* were identified in all patients. In C1-INH-HAE type I, we identified 19 different mutations, including 6 missense mutations, 6 nonsense mutations, 2 small deletions, 1 small insertion, 2 splicing defects and 2 large deletions. Two of the mutations (c.300C>T and c.1184_1185insTA) are reported here for the first time. All C1-INH-HAE type II patients from three families harboured the same substitution (c.1396C>T). Based on the type of mutation identified in the *SERPING1* gene, patients were divided into two groups: group 1 (nonsense, frameshift, large deletions/insertions, splicing defect, and mutations at Arg444) or group 2 (missense, excluding mutations at Arg444). Significant differences were found in the clinical severity score (P = 0.005), prevalence of laryngeal (P = 0.040) and facial (P = 0.013) oedema, and long-term prophylaxis (P = 0.023) between the groups with different types of mutations. Because our population consisted of related subjects, differences in the severity score between mutation groups were further confirmed using the generalized estimating equation (P = 0.038). Our study identified 20 different disease-causing mutations, including two novel mutations, in all C1-INH-HAE patients, highlighting the heterogeneity of mutations in the *SERPING1* gene. Furthermore, it appears that mutations with a clear effect on C1-INH function might be responsible for a more severe disease phenotype.

## Introduction

Hereditary angioedema due to C1 inhibitor deficiency (C1-INH-HAE) is a rare autosomal dominant disease characterized by recurrent and potentially life-threatening swelling of the skin and upper respiratory tract and abdominal pain. It has an estimated prevalence of one case per 50,000 persons [1–4]. This rare disease is caused by mutations affecting the C1 inhibitor (C1-INH) gene, *SERPING1*. C1-INH-HAE can be further divided into two subtypes. C1-INH-HAE type I results in low levels of C1-INH and accounts for 85% of cases. C1-INH-HAE type II results in normal levels of ineffective C1-INH and accounts for 15% of cases [1–3]. C1-INH is a serine protease inhibitor, and its main function is preventing inappropriate or excessive activation of the complement system. In addition to the classical pathway, C1-INH also controls the lectin complement pathway and contact, fibrinolytic and coagulation cascades, thus representing a key regulator of several immune and inflammatory pathways [1,2].

The *SERPING1* gene (OMIM #606860) is located on chromosome 11.q12-q13.1, and it consists of eight exons. The first exon contains 38 bp of noncoding sequence, and the second has a 22 bp-long signal peptide before the first methionine; the seven introns distributed over 17 kb contain 17 repetitive *Alu* sequences [1,5]. To date, more than 400 different mutations distributed across the entire *SERPING1* gene have been described, ranging from nucleotide substitutions to small insertions and deletions to large deletions and duplications (HAEdb, http://www.hae.enzim.hu) [6]. *De novo* mutations in *SERPING1* account for approximately 25% of cases with C1-INH-HAE [1,7].

The aim of this study was to analyse and report the mutational findings in a cohort of 40 patients and four asymptomatic subjects from 27 Serbian families. This is the first report investigating the molecular causes of C1-INH-HAE in Serbia. Furthermore, due to high *SERPING1* gene and C1-INH-HAE clinical variability, we investigated the genotype-phenotype relationship to test the hypothesis that nonsense mutations, frameshift mutations, large deletions/insertions, splicing defects, and mutations at Arg444 might be responsible for a more severe disease phenotype in comparison to missense mutations, with the exception of mutations at Arg444.

## Materials and Methods

### Patients

The patients were diagnosed and recruited during a 20-year period (1995–2015) at the Clinical Center of Serbia (Clinic of Allergy and Immunology), which serves a population of 4 million inhabitants. The Clinical Center of Serbia is a tertiary medical centre responsible for the management of rare diseases in central, northern and western parts of Serbia. Forty patients with C1-INH-HAE, including 4 asymptomatic subjects, from 27 unrelated Serbian families were recruited for genetic analysis. The diagnosis of C1-INH-HAE was established in the presence of clinical (subcutaneous angioedema, abdominal pain, laryngeal oedema) and laboratory criteria (C1-INH antigenic levels, C1-INH function) and followed up with positive family history, as proposed in the guidelines for the diagnosis of C1-INH-HAE [1–4]. This study was conducted in accordance with the amended Declaration of Helsinki and was approved by the Ethics Committee of the Clinical Center of Serbia (Protocol number 14/6), and all participants gave their written informed consent.

### Clinical severity score

The clinical severity score was calculated based on the age of disease onset (0–5 years = 3 points, 6–10 years = 2 points, 11–20 years = 1 point, >20 years = 0 points), number of organs affected (skin oedema = 1 point, painful abdominal oedema = 2 points, laryngeal oedema = 2 points, other clinical manifestations = 1 point) and long-term prophylaxis (long term prophylaxis = 1 point). Clinical severity score was expressed with values from 0 to 10 as proposed by Bygum et al. [8].

### Complement testing

Serum protein concentrations of C1-INH (normal range: 0.20–0.35 g/l) and C4 (normal range: 0.16–0.31 g/l) (Siemens, Marburg, Germany) were quantified by means of radial immunodiffusion and C1-INH function (C1-INH function levels ≤ 40% of normal are considered decreased) were measured using an enzyme immunoassay (Quidel Corporation, California, USA) in accordance with the manufacturer's instructions.

### Genotyping

Genomic DNA was extracted from EDTA-containing whole blood samples using a QIAamp DNA Blood Mini Kit (Qiagen, Hilden, Germany) according to the manufacturer’s instructions. The detection of *SERPING1* mutations in the promoter, noncoding exon 1, the 7 coding exons and exon-intron boundaries was performed as previously described [3,9]. To identify mutations, all sequences were compared with the *SERPING1* reference sequence in GenBank (GenBank accession number X54486.1). *SERPING1* variations were numbered in two ways: the traditional genomic numbering considers the first nucleotide of exon 1 to be number one [5], whereas the systematic cDNA numbering considers the first nucleotide (A) of the initiation methionine (ATG) of the cDNA sequence (GenBank accession number NM_000062.2) to be nucleotide number one. For the protein amino acid positions, the study used traditional numbering based on the mature protein of 478 amino acids, counting the first 22 amino acids of the N-terminal residue of the signal peptide in negative numbers. In negative cases in which the mutation could not be identified by sequencing, samples were further analysed for large deletions/duplications using multiplex ligation-dependent probe amplification (MLPA). The SALSA MLPA P243-A2 SERPING1 kit (MRC-Holland, The Netherlands) was used, and data were analysed with Coffalyser MLPA data analysis software (MCR-Holland).

### Mutation grouping

Based on the type of mutation identified in the *SERPING1* gene, patients were divided into two groups, slightly modified from those previously described [10–13]: group 1 (nonsense, frameshift, large deletions/insertions, splicing defect, and mutations at Arg444) or group 2 (missense, excluding mutations at Arg444).

### Statistical Analysis

Data distribution was evaluated by the D’Agostino–Pearson test. Parametric statistics (unpaired t-tests) were used on normally distributed data, and non-parametric statistics (the Mann–Whitney) were used if the distribution deviated from normal. Two-sided Fisher's exact test was used to calculate the significance of the differences in patient’s clinical characteristics (oedema on different body parts and the need for long-term prophylaxis) between the two mutation groups. Spearman’s rank correlation test was used to analyse the correlation between the two mutation groups and the patient’s clinical characteristics. A generalized estimating equation (GEE) with robust covariate matrix and exchangeable correlation structure was implemented to model the association of response variables (clinical severity score, age at onset of symptoms, oedema on different body parts and need for long-term prophylaxis) with explanatory variables (mutation group, age, gender) because our patient population consisted of related subjects. Clinical severity score and age at disease onset were modelled as continuous variables in linear GEE models, whereas oedema on different body parts and the need for long-term prophylaxis were entered as binary variables in the logistic GEE model. GraphPad Prism 5.0 software (San Diego, CA, USA) and R [14] with its affiliated packages [15] were used for statistical analysis. A P-value of less than 0.05 was considered statistically significant.

## Results and Discussion

### Clinical details

Thirty-nine (89%) patients, among them four asymptomatic subjects, from twenty-four families were diagnosed with C1-INH-HAE type I, and five (11%) patients from three families were diagnosed with C1-INH-HAE type II. Clinical data of the recruited patients are presented in Table 1. Three youths (7, 13 and 14 years old) and one adult (58 years old) were asymptomatic, although their complement tests and mutation analysis were consistent with C1-INH-HAE diagnosis. Asymptomatic patients were excluded from further clinical and genotype-phenotype association analyses. All patients had reduced serum C4 (range <0.05–0.14 g/l). C1-INH-HAE type I patients had highly reduced concentrations of C1-INH in serum (range <0.05–0.09 g/l), whereas patients with C1-INH-HAE type II had increased levels of C1-INH (range 0.60–>0.73 g/l). Median C1-INH function for both C1-INH-HAE types was 9% (range 0–53%).

**Table 1:** Clinical data of Serbian patients with C1-INH-HAE.

Patient	Family	Age (years)	Gender	Age at onset of symptoms	C1-INH-HAE type	Clinical severity score	Skin oedema	Facial oedema	Abdominal oedema	Laryngeal oedema	Prophylactic treatment	Family history
1	1	31	F	3	Type I	9	+	+	+	+	Danazol/Tranexamic Acid	−
2	2	37	F	6	Type I	8	+	+	+	+	Tranexamic Acid	−
3	3	48	F	3	Type I	7	+	+	+	−	Danazol	+
4	3	46	F	1	Type I	9	+	+	+	+	Tranexamic Acid	+
5	3	25	F	5	Type I	7	+	+	+	−	Tranexamic Acid	+
6	4	51	F	14	Type I	2	+	−	−	−	None	+
7	4	48	M	13	Type I	8	+	+	+	+	Danazol	+
8	4	32	F	4	Type I	8	+	+	+	+	None	+
9	4	30	F	13	Type I	6	+	−	+	+	None	+
10	4	26	M	24	Type I	1	+	−	−	−	None	+
11	4	18	M	14	Type I	4	+	−	+	−	None	+
12	4	17	M	16	Type I	2	+	−	−	−	None	+
13	4	7	M	ND	Asympt	ND	ND	ND	ND	ND	None	+
14	5	45	F	14	Type I	7	+	+	+	+	Danazol/Tranexamic	−
15	6	64	M	6	Type II	8	+	+	+	+	Danazol	+
16	6	31	M	3	Type II	9	+	+	+	+	Danazol	+
17	7	29	M	14	Type II	7	+	+	+	+	Danazol	−
18	8	25	F	1	Type I	6	+	+	+	−	None	−
19	9	73	M	12	Type I	7	+	+	+	+	Danazol	+
20	10	62	M	13	Type I	7	+	+	+	+	Danazol	+
21	11	53	F	3	Type I	9	+	+	+	+	None	−
22	12	63	F	24	Type I	5	+	+	+	+	None	+
23	13	68	F	2	Type I	8	+	+	+	+	None	−
24	14	54	F	20	Type I	4	+	+	−	−	Danazol	+
25	15	40	F	9	Type I	6	+	+	−	+	None	−
26	16	50	F	10	Type II	6	+	+	−	+	None	+
27	16	28	F	8	Type II	6	+	+	−	+	Tranexamic Acid	+
28	17	76	F	15	Type I	7	+	+	+	+	Danazol	+
29	17	30	M	4	Type I	9	+	+	+	+	Danazol	+
30	17	27	M	6	Type I	8	+	+	+	+	Danazol	+
31	18	25	M	7	Type I	6	+	+	+	−	Danazol	−
32	19	59	M	5	Type I	9	+	+	+	+	Danazol/Tranexamic Acid	−
33	20	58	M	6	Type I	6	+	+	−	+	Danazol	+
34	20	28	M	13	Type I	4	+	+	−	−	Danazol	+
35	21	57	M	6	Type I	8	+	+	+	+	Danazol	+
36	22	38	M	7	Type I	9	+	+	+	+	None	−
37	23	27	M	15	Type I	6	+	+	+	+	None	+
38	24	39	M	18	Type I	7	+	+	+	+	None	−
39	24	13	M	ND	Asympt	ND	ND	ND	ND	ND	None	+
40	25	49	F	25	Type I	4	+	+	+	−	None	+
41	26	42	M	17	Type I	8	+	+	+	+	Tranexamic Acid	+
42	27	58	M	ND	Asympt	ND	ND	ND	ND	ND	None	−
43	27	26	F	7	Type I	9	+	+	+	+	Tranexamic Acid	+
44	27	14	M	ND	Asympt	ND	ND	ND	ND	ND	None	+

Asympt: Asymptomatic; ND: Not determined

The prevalence of C1-INH-HAE is estimated to be approximately one case per 50,000 persons without major ethnic or gender differences [2,4]. The calculated prevalence derived from a clinical centre serving a population of 4 million inhabitants in Serbia is approximately 1:100,000, which is similar to that in Slovenia [9] and Spain [16–18], but slightly lower than that in Denmark, Norway, Sweden and Italy [8, 19–22], where the calculated prevalence is 1:65,000 to 1:70,000 inhabitants. These data suggest that this rare disease may still be underdiagnosed in some countries. The frequencies of C1-INH-HAE type I (89%) and type II (11%) are in agreement with those previously reported in other European countries [10, 13, 16, 23–25].

The mean age at the onset of clinical symptoms was 10 years (range 1–25 years). Major symptoms were skin oedema in all 40 patients (100%), followed by facial oedema in 35 patients (88%), abdominal oedema in 31 patients (78%) and laryngeal oedema in 29 patients (73%). Twenty five (63%) patients had received long-term prophylaxis with attenuated androgen danazol and/or tranexamic acid for a length of time, and nine of them had received these treatments continuously for more than ten years. Thirteen patients (30%) had no known family history of angioedema. The median clinical severity score based on age of disease onset, organs affected, and long-term prophylaxis was 7.0 (range 1–9).

In our cohort, the delay from symptom onset to establishment of correct C1-INH-HAE diagnosis was highly variable, ranging from less than 1 year in five patients to as high as 38 years in one patient. The median delay was 11 years and was comparable (P = 0.449) in patients diagnosed with C1-INH-HAE type I (11 years) or type II (6 years).

### Genetic analysis

In all 40 patients with C1-INH-HAE and in the four asymptomatic subjects from 27 unrelated families, a disease-causing mutation in the *SERPING1* gene was identified, whereas no mutations were present in healthy relatives and controls.

**Fig 1:**
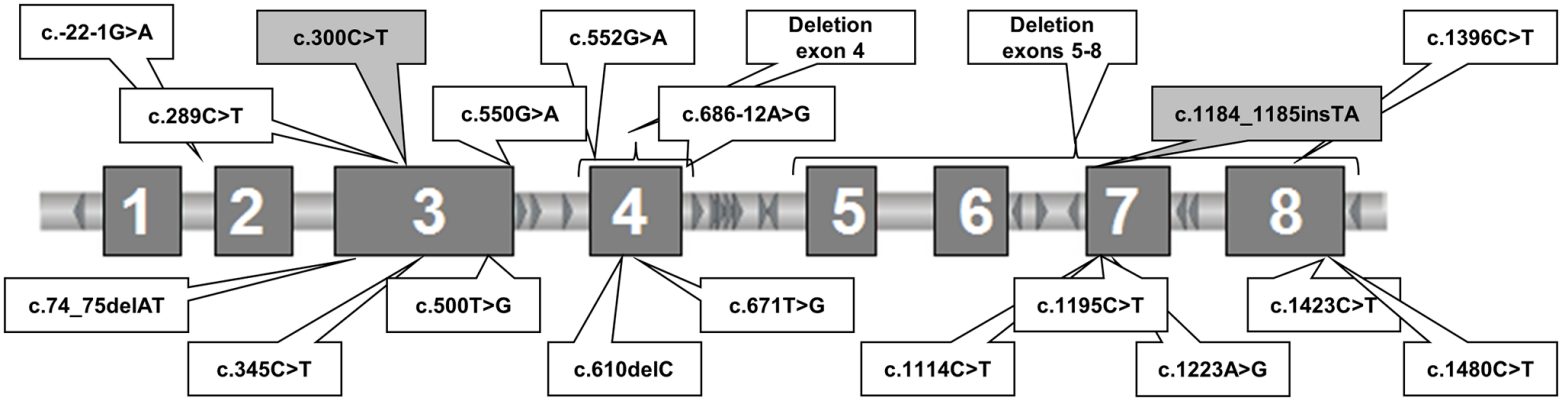
Mutations identified in the *SERPING1* gene in Serbian families with hereditary angioedema due to C1 inhibitor deficiency. Boxes showing the novel mutations identified are shaded in grey.

We identified 20 different mutations, all in the heterozygous state, among them 7 missense mutations, 6 nonsense mutations, 2 small deletions and 1 small insertion causing a frameshift, 2 splicing defects, and 2 large deletions, one of exon 4 and one of exons 5 to 8. Twenty-five families carried known mutations, whereas two mutations were reported for the first time (Fig 1, Table 2).

**Table 2:** Mutations found in Serbian patients with C1-INH-HAE.

Traditional genomic numbering	cDNA numbering	Position	Predicted effect on protein (traditional numbering)	No. of families	No. of patients	Reference	Mutation group^1^
g.8313A>G	c.686−12A>G	Intron 4	Splicing defect	1	1	10	1
g.4471T>G	c.671T>G	Exon 4	Ile202Ser	1	1	17	2
**g.2445C>T**	**c.300C>T**	**Exon 3**	**Gln79Stop**	**1**	**3**	**This study**	**1**
g.14196C>T	c.1195C>T	Exon 7	Pro377Ser	4	12	15	2
g.2694G>A	c.550G>A	Exon 3	Splicing defect or Gly162Arg	2	2	14	2
g.16788C>T	c.1396C>T	Exon 8	Arg444Cys	3	5	24	1
g.2644T>G	c.500T>G	Exon 3	Met145Arg	2	2	18	2
g.2212_2213delAT	c.74_75delAT	Exon 3	Frameshift	1	1	21	1
g.14244A>G	c.1223A>G	Exon 7	Asp386Gly	1	1	19	2
g.2490C>T	c.345C>T	Exon 3	Gln94Stop	1	1	7	1
Deletion of exons 5 to 8				1	1	18	1
g.4351G>A	c.552G>A	Exon 4	Gly162Glu	1	1	16	2
**g.14185_14186insTA**	**c.1184_1185insTA**	**Exon 7**	**Frameshift**	**1**	**3**	**This study**	**1**
g.564G>A	c.-22-1G>A	Intron 1	Splicing defect	1	1	14	1
g.4400delC	c.610delC	Exon 4	Frameshift	1	1	14	1
g.17997C>T	c.1423C>T	Exon 8	Gln453Stop	1	1	13	1
Deletion of exon 4				1	1	17	1
g.15297C>T	c.1114C>T	Exon 7	Gln350Stop	1	2	17	1
g.18054C>T	c.1480C>T	Exon 8	Arg472Stop	1	1	14	1
g.3615C>T	c.289C>T	Exon 3	Gln75Stop	1	3	12	1

New mutations are in boldface type.

^1^Group 1 (nonsense, frameshift, large deletions/insertions, splicing defect, and mutations at Arg444) or group 2 mutations (missense mutations, excluding mutations at Arg444).

Six missense mutations were identified in ten C1-INH-HAE type I families: two in exon 3, Met145Arg (c.500T>G) and Gly162Arg (c.550G>A); two in exon 4, Gly162Glu (c.552G>A) and Ile202Ser (c.671T>G); and two in exon 7, Pro377Ser (c.1195C>T) and Asp386Gly (c.1223A>G). All missense mutations were previously described [16, 23, 24, 26–28]. The mutation c.550G>A affects the last base of exon 3, simultaneously altering the splicing and the amino acid sequence (Gly162Arg) [7,16,17,23,24,26]. Missense mutation c.1195C>T was present in four unrelated families with 12 affected individuals altogether, whereas mutations c.500T>G and c.550G>A were each present in two unrelated patients. All other missense mutations identified in C1-INH-HAE type I were distinct for each family.

Among 6 nonsense mutations identified in three C1-INH-HAE type I families, 5 were already reported: Gln75Stop (c.289C>T), Gln94Stop (c.345C>T), Gln350Stop (c.1114C>T) Gln453Stop (c.1423C>T), and Arg472Stop (c.1480C>T) [7,12,13,16,26]. One novel mutation in exon 3 Gln79Stop (c.300C>T) was identified in three affected members of the same family, whereas this mutation was absent in a healthy relative. The newly identified nonsense mutation causes a premature termination of the protein at amino acid 79.

Two previously described mutations, c.74_75delAT and c.610delC [26,29], detected in two patients with C1-INH-HAE type I caused a frameshift. The patient with the deletion c.74_75delAT was previously reported [29]. In three affected family members, a novel insertion of two nucleotides in exon 7 (c.1184_1185insTA) was identified. This novel mutation causes a frameshift and premature termination of the protein two amino acids downstream of the mutation.

In our cohort of patients, two previously described mutations causing splicing defects were identified, specifically, c.-22-1G>A in intron 1 and c.686-12A>G in intron 4 [10,26] in two families with C1-INH-HAE type I.

By MLPA analysis, two large deletions were detected in two patients with C1-INH-HAE type I: the first was a deletion of exon 4, and the second was a deletion of exons 5–8. Large deletions affecting exon 4 as well as exons 5–8 have previously been described in C1-INH-HAE patients [7,16,24,25,30].

Two families each with two affected members and one sporadic patient with C1-INH-HAE type II harboured the same well-known substitution affecting the arginyl residue at the reactive centre in exon 8, Arg444Cys, c.1396C>T [1, 6–13, 16, 17, 23–31]. Those five patients had a normal C1-INH level but reduced C1-INH activity because mutations at this position alter the target protease recognition site of this protein.

The identified substitutions and small deletions were distributed throughout the entire *SERPING1* gene; specifically, 6% were located in the region from the promoter to intron 2, 33% in exon or intron 3, 22% in exon or intron 4, 22% in exon or intron 7 and 17% in exon 8. Interestingly, although the mutations were distributed throughout the entire *SERPING1* gene, we detected a substantially higher frequency of mutations located in exon or intron 4 in comparison to other reports [6,13,17,24]. In our cohort, 22% of all detected mutations were located in this region, whereas the previously reported frequencies were between 7 and 11% [6,13,17,24].

### Genotype-phenotype association

To address a possible association between the type of mutation and clinical presentation of the disease, patients were divided into two groups based on mutation type, as described previously [10–13]. In group 1, we included nonsense and frameshift mutations, large deletions/insertions, splicing defects, and mutations at Arg444. Missense mutations, excluding mutations affecting the arginyl residue at the reactive centre in exon 8 (Arg444), were grouped together (group 2). This grouping of different types of mutations is a combination of the grouping previously proposed [10–13] because some studies [10,11,13] grouped together all missense mutations that are likely to allow stable transcripts, whereas Xu *et al*., 2012 have proposed grouping of missense mutations affecting the reactive centre at Arg444 with nonsense and frameshift mutations [12]. There are two limitations of this grouping method [12]: first, splicing defects were excluded from analysis and, second, lack of testing for large deletions/insertions resulted in an inability to categorize those types of mutations. To overcome those limitations, we grouped together all other mutations with a clear effect on C1-INH function, including missense mutations involving the reactive centre at Arg444 in group 1. However, we grouped together only missense mutations for which it was difficult to determine the impact on the C1-INH function in group 2. Group 1 mutations cause either premature termination of mRNA translation or alter the reading frame, resulting in truncated proteins or unstable mRNA transcripts that are removed through the nonsense mediated mRNA decay pathway [32,33]. Two types of evaluations were carried out. Initially, we analysed only 1 patient, the index case, from each family. We found that patients from group 1 had a higher clinical severity score (mean: 7.6 vs 6.1; P = 0.049) and more often had laryngeal oedema (94% (15/16) vs 55% (5/11); P = 0.027; Table 3). Afterwards, we performed the same analysis in the whole group of patients and found that patients with group 1 mutations tended to have higher CSS (mean: 7.5 vs 5.7; P = 0.005) and more often had laryngeal (86% (19/22) vs 56% (10/18); P = 0.040) and facial (100% (22/22) vs 72% (13/18); P = 0.013) oedema (Table 3). There was a significant correlation between the type of mutation and CSS (P = 0.009; r_s_ = −0.409) and the presence of laryngeal (P = 0.030; r_s_ = −0.343) and facial (P = 0.007; r_s_ = −0.418) oedema. No association between the different types of mutations and disease onset or oedema on other parts of the body was found. Patients with group 1 mutations were more often on long-term prophylaxis (77% (17/22) vs 39% (7/18); P = 0.023), and a significant correlation between the type of mutation and prophylactic treatment (P = 0.013; r_s_ = −0.390) was observed (Table 3). Because our patient population consisted of related subjects, we performed GEE with CSS as a continuous variable and found that patients with group 1 mutations had a higher CSS in comparison to patients from group 2 (P = 0.038, β coefficient = 1.23, unadjusted; P = 0.030, β coefficient = 1.35, adjusted for gender and age; Table 3). Interestingly, the most common mutation detected in our cohort, c.1195C>T, which was present in four families, was associated with a less severe clinical phenotype, with a mean severity score of 4.7 and a mean disease onset at 14 years. Because we did not have laboratory data of C4 and C1-INH concentrations and functions before prophylaxis therapy for all patients, we did not perform analysis for the association between the type of mutation and complement factors.

**Table 3:** Genotype-phenotype association analysis in C1-INH-HAE patients from Serbia.

	Unrelated patients				All patients				
	Group 1 (N = 16)	Group 2 (N = 11)	P value		Group 1 (N = 22)	Group 2 (N = 18)	P value		
			t-test/Fisher	Spearman			t-test/Fisher	Spearman	GEE
Age at onset of symptoms, years, mean (SD)	10.4 (7.0)	9.7 (7.0)	0.815	0.736	9.5 (6.4)	10.9 (6.8)	0.495	0.601	0.937
Clinical severity score, mean (SD)	7.6 (1.6)	6.1 (2.1)	0.049	0.050	7.5 (1.4)	5.7 (2.4)	0.005	0.009	0.038
Skin oedema, %	100%	100%	NA	NA	100%	100%	NA	NA	NA
Facial oedema, %	100%	91%	0.407	0.235	100%	87%	0.013	0.007	NA
Abdominal oedema, %	88%	73%	0.371	0.351	86%	67%	0.253	0.145	0.347
Laryngeal oedema, %	94%	55%	0.027	0.015	86%	56%	0.040	0.030	0.062
Prophylactic treatment, %	69%	45%	0.264	0.242	77%	39%	0.023	0.013	0.108

Group 1 mutations: nonsense, frameshift, large deletions/insertions, splicing defect, and mutations at Arg444.

Group 2 mutations: missense mutations, excluding mutations at Arg444.

GEE: Generalized estimating equations with robust covariate matrix and exchangeable correlation structure; NA: Not applicable; SD: standard deviation; Spearman: The Spearman’s rank correlation test; t-test/Fisher: unpaired t-tests or two-sided Fisher's exact test as appropriate.

The analysis of the association between *SERPING1* mutation types and clinical presentation of the disease revealed that group 1 mutations were associated with a more severe clinical phenotype in comparison with group 2 mutations. Significant differences were found, especially in clinical severity score but also in the prevalence of laryngeal and facial oedema, as well as the need for long-term prophylaxis, between the different groups of mutations. Our results indicate that different types of mutations might be responsible for the high variability of C1-INH-HAE clinical expression, which is in agreement with two reports that have suggested an association of disease severity and disease onset with mutation type [11,13]. However, several other studies have failed to demonstrate the genotype-phenotype association in C1-INH-HAE patients [8,12,17,34]. Moreover, new studies have demonstrated that several other disease modifying factors, such as polymorphism rs1801020 in *F12* [11,35], play important roles in determining clinical variability of C1-INH-HAE. Therefore, we propose that our modified categorization of mutations into two groups should be adopted in further genotype-phenotype association analyses because at least part of the discrepancy between our findings and previous studies might be due to the different categorization of mutations. However, we are aware of the limitations of our study, namely, low sample size and the inclusion of relatively small number of mutations. If our results are confirmed in additional populations and larger sample sizes, the detection of and information about mutation type could contribute to improvements in the early management of this potentially life-threatening oedema already in infants, before the appearance of clinical symptoms.
